# Mental Fatigue in High School Students Through Spanish Physical Education Teachers’ Perceptions of Causes, Consequences, and Reduction Strategies: A Survey Study

**DOI:** 10.3390/healthcare14070960

**Published:** 2026-04-06

**Authors:** Francisco Javier Roldán-Ramos, Juan de Dios Benítez-Sillero, Ana Rodríguez-Cano, Javier Raya-González

**Affiliations:** Research Group on Sport and Physical Education for Personal and Social Development (GIDEPSO), Faculty of Education Sciences and Psychology, University of Córdoba, 14004 Córdoba, Spain; l72roraf@uco.es (F.J.R.-R.); eo1besij@uco.es (J.d.D.B.-S.); rayagonzalezjavier@gmail.com (J.R.-G.)

**Keywords:** fatigue, burnout, youth, teachers, views

## Abstract

**Background/Objectives**: Mental fatigue in adolescents is a growing concern in educational contexts, positioning physical education (PE) teachers as key agents in designing effective mitigation strategies. This study examined the perceptions of Spanish high school PE teachers regarding the causes, consequences, and potential countermeasures for students’ mental fatigue. **Methods**: A total of 116 in-service teachers (81 males and 35 females; mean teaching experience 7.8 ± 5.3 years) from 12 autonomous communities throughout Spain completed a comprehensive 34-item electronic questionnaire. The instrument assessed the perceived existence, etiology, and outcomes of mental fatigue through multiple-choice, dichotomous (yes/no), and five-point Likert scale questions, with particular attention given to the role of physical activity (PA) in symptom alleviation. A quantitative frequency analysis was conducted to examine the data. **Results**: The main findings reveal a strong consensus among the teachers (77.6% to 87.9%) on the prevalence of mental fatigue, with its primary causes attributed to academic pressure and sedentarism. The consequences were identified as increased irritability and reduced cognitive performance. The teachers overwhelmingly endorsed moderate intensity PA as the most effective countermeasure. However, a significant gap was identified between this theoretical awareness and the systematic implementation of targeted strategies within schools. **Conclusions**: These results underscore the critical need for professional development programs and structural support to translate teacher knowledge into practical intervention, suggesting important directions for future research.

## 1. Introduction

In recent decades, mental fatigue (MF) in adolescents has emerged as a critical concern in educational settings, exacerbated by rising academic pressures, social dynamics, and prolonged exposure to digital technologies [1]. In Spain, as in other European countries, the COVID-19 pandemic further amplified these challenges, disrupting physical activity (PA) patterns, sleep hygiene, and social interactions among youth [2]. Mental fatigue, defined as a psychobiological state characterized by feelings of tiredness, exhaustion, reduced energy, or a diminished desire to continue with a task [3], not only impairs academic performance but is also linked to health risks of psychological disorders such as anxiety and depression [4]. Moreover, it correlates with reduced motivation to engage in physical and social activities, creating a cyclical decline in overall well-being [5]. It must be acknowledged that mental fatigue can manifest differently depending on the type of cognitive load; therefore, we have clarified in the Introduction the distinction between acute mental fatigue, which arises after intense but brief cognitive tasks, and chronic forms, such as school-related burnout resulting from an accumulated overload. This distinction also highlights the role of physical education teachers in mitigating both immediate and long-term manifestations of mental fatigue in adolescents.

The modern school environment exhibits distinct characteristics that contribute to MF [6]. Rigid schedules and curricular structures frequently require students to engage in prolonged sedentary periods involving passive, cognitively demanding tasks [7]. These demands are compounded by significant academic pressure stemming from high workload, task complexity, and performance expectations [8,9,10]. Additional stressors include external pressures from educators, parents, and institutional demands [11], as well as motivational and social challenges [12]. Physical Education (PE) represents a promising avenue for mitigating these effects, offering both active lifestyle promotion [13] and demonstrated benefits for cognitive function, stress reduction, and emotional resilience [14]. School-based interventions, such as physically active learning, movement breaks, and active recesses, have been successfully implemented to increase adolescent PA, demonstrating positive outcomes [15,16]. However, implementation barriers persist, particularly regarding curricular conflicts, inadequate PE program integration, and limited stakeholder engagement [17]. Overcoming these barriers is essential for the effective, sustained implementation of PA interventions that may reduce adolescent MF in educational settings.

In this context, PE teachers serve as pivotal agents in implementing PA-based strategies, helping to overcome some of these identified barriers [18]. Thus, their insights into the manifestations, causes, and consequences of MF, as well as their perceptions of the mitigating role of PA, are critical for designing effective school-based interventions [19]. However, to date, this valuable information remains unexplored, as the predominant research has focused on student self-reports or physiological markers of fatigue [20]. This approach has been successfully used in other contexts [21,22], facilitating more specific knowledge of the target topic. Therefore, understanding PE teachers’ perceptions of MF in adolescents would provide a comprehensive understanding not only of the current state of knowledge on this topic but would also reveal gaps between the literature and actual educational practice, while assisting in the design and implementation of effective future measures.

To address the identified research gaps, this study examined the Spanish high school PE teachers’ perceptions of student MF, including its causes, consequences, and potential mitigation strategies. We hypothesized that PE teachers recognize MF among their students and understand PA’s role in addressing it, and PE teachers are knowledgeable about school-based PA strategies for MF reduction, but the implementation of these strategies remains inconsistent in practice.

## 2. Materials and Methods

### 2.1. Participants

One hundred and sixteen high school PE teachers (7.8 ± 5.3 years of experience) voluntarily participated in this study. The PE teachers involved in the study represented 12 devolved regions in Spain. Out of the total number of participants, 81 were male PE teachers, and 35 were female PE teachers. Also, 100 PE teachers worked in public schools, and 16 worked in private schools. The participants were recruited via the authors’ professional networks and social media platforms. The sample size was maximized through chain sampling, whereby participants were encouraged to invite relevant people within their networks. All participants were informed about the aims and procedures of the study, and they gave their electronic informed consent prior to participation. The study protocol and survey adhered to the principles of the Declaration of Helsinki and were approved by the ethics committee of the University of Córdoba (Code: CEIH-24-47).

### 2.2. Experimental Approach to the Problem

The survey study was conducted through an electronic questionnaire (Google Drive, California, US) that was completed by PE teachers. A total of 34 questions were included in the electronic questionnaire, which were grouped into 4 topics: (1) existence of MF in students; (2) causes of MF in students; (3) consequences of MF in students; and (4) the role of PA on the MF attenuation. Specifically, the electronic questionnaire was composed of 2 multiple-choice questions; 7 yes/no questions; and 24 questions based on a five-point Likert scale (strongly disagree, disagree, neither agree nor disagree, agree, and strongly agree). This combination of question types allowed the participants to report their level of agreement regarding each statement. Finally, one open-ended question was also included, allowing PE teachers to provide more details about the topic. The questionnaire items were developed based on the evidence reported in previous studies on the topic [7,8,9,10,19]. Additionally, a pilot test with 20 PE teachers was conducted to ensure the clarity and relevance of the items.

### 2.3. Quantitative Analysis

The frequencies were determined for each close-ended question or Likert-type scale response, with many of the responses also presented as frequency plots. All the participants were included in the final analysis.

## 3. Results

### 3.1. Existence of Mental Fatigue in Students

A total of 101 PE teachers acknowledged awareness of MF among students, while 12 denied its existence, and three responded, “don’t know/no answer.” Notably, 96 teachers reported the ability to detect signs of MF, whereas 13 stated they were unable to identify such indicators. Seven respondents selected “don’t know/no answer” for this question. Finally, 112 teachers observed fluctuations in MF across the academic year, with only two reporting consistent levels.

### 3.2. Causes of Mental Fatigue in Students

**Figure 1:**
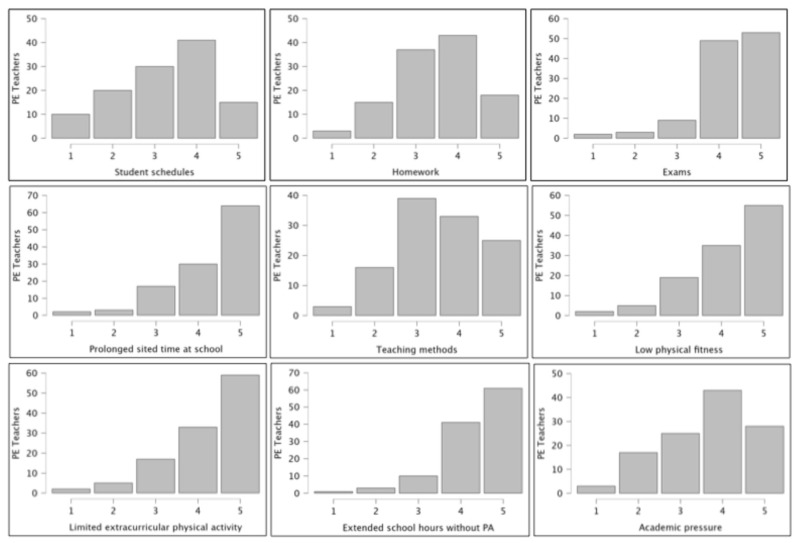
The physical education teachers’ perceptions regarding the causes of mental fatigue.

Figure 1 graphically represents PE teachers’ perceptions regarding causes of MF. Approximately half of the respondents agreed or strongly agreed that students’ schedules (56/116; 48.3%), homework (61/116; 52.6%), teaching methods (58/116; 50.0%), and academic pressure (71/116; 61.2%) contribute to MF. In contrast, a strong majority identified exams (102/116; 87.9%), prolonged sited time at school (94/116; 81.0%), low physical fitness (90/116; 77.6%), limited extracurricular PA (92/116; 79.3%), and extended school hours without PA (102/116; 87.9%) as significant causative factors.

### 3.3. Consequences of Mental Fatigue in Students

**Figure 2:**
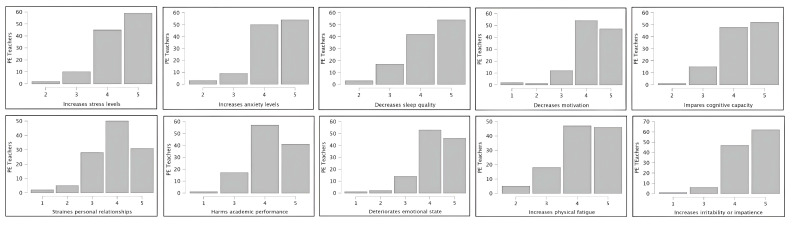
The physical education teachers’ perceptions regarding the consequences of mental fatigue.

The PE teachers’ perceptions regarding the consequences of MF are presented in Figure 2. The vast majority perceived significant negative impacts, with over 85% of the respondents agreeing or strongly agreeing that MF leads to increased stress (104/116), heightened anxiety (104/116), and reduced motivation (101/116). Additionally, more than 80% of teachers linked MF to impaired cognitive capacity (100/116), worsened academic performance (98/116), and deterioration in emotional state (99/116). Notably, increased irritability or impatience was the most widely endorsed consequence (109/116, 94% agreement). Other notable effects included declines in sleep quality (96/116), strained personal relationships (81/116), and elevated physical fatigue (93/116).

### 3.4. The Role of Physical Activity in Mental Fatigue Attenuation

The survey revealed a strong consensus among PE teachers regarding the strategies to mitigate students’ MF. When asked about MF’s general modifiability, 106 respondents (91.4%) affirmed its potential for positive influence, versus two (1.7%) who denied this possibility and eight (6.9%) who remained undecided. Specifically, a substantial majority (100/116, 86.2%) agreed that MF could be reduced through PA strategies, while only three (2.6%) disagreed and three (2.6%) were uncertain. Similarly, 106 teachers (91.4%) believed that increasing physical fitness could reduce MF, contrasted by five (4.3%) who disagreed and five (4.3%) who expressed uncertainty. Regarding practical implementation, 80 teachers (69.0%) reported actively applying either general or specific strategies to reduce student MF, while 25 (21.6%) did not employ such methods, and nine (7.8%) were unsure about their current practices.

**Figure 3:**
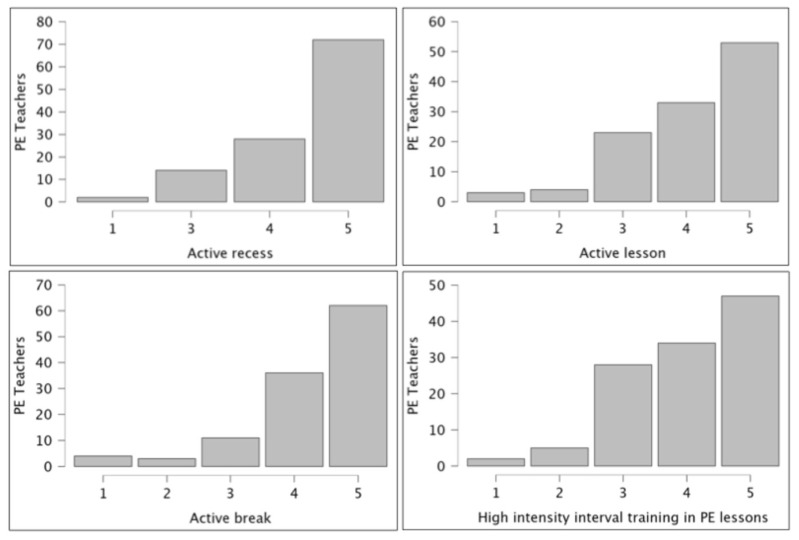
The physical education teachers’ perceptions regarding the effectiveness of school-based physical activity strategies for mental fatigue.

Figure 3 shows PE teachers’ assessments of various school-based PA strategies for reducing student MF. Among the evaluated interventions, active recess programs received the highest effectiveness rating (516 points), followed by active breaks (497 points) and active lessons (477 points). In contrast, high-intensity PA was perceived as the least effective approach (467 points), sharing this rating with unspecified “other strategies” (404 points). These findings suggest that PE teachers view moderate, integrated physical activities (particularly recess-based interventions) as more effective for MF reduction than either high-intensity exercise or alternative unspecified methods.

## 4. Discussion

This study examined Spanish high school PE teachers’ perceptions of student MF, encompassing its perceived causes, consequences, and potential mitigation strategies. This research represents the first investigation to specifically analyze PE teachers’ perspectives on this pertinent issue, thereby contributing valuable insights to facilitate the design of comprehensive, context-specific strategies, predominantly grounded in PA, to mitigate the adverse effects of MF. The principal findings indicate that PE teachers recognize the presence of MF among their students, identify its key causes and consequences, and acknowledge the role of PA (including specific examples) in addressing it. However, the implementation of these targeted strategies within the school environment by PE teachers remains limited.

### 4.1. Existence of Mental Fatigue in Students

Being aware of the existence of substantial levels of MF in students is the first step in being able to intervene [23]. Our quantitative findings empirically validate this premise, since 101 participants (87.0%) acknowledged prevalent MF among students, contrasting with 12 dissenters (10.3%) and three respondents (2.6%) who declined to take a stance. This distribution not only corroborates those reported by Earl et al. [1], who documented the escalation of MF in adolescent student populations, but further demonstrates teachers’ progressive engagement in both identifying and mitigating its academic and behavioral impacts [24]. Furthermore, the identification of MF indicators is essential for timely intervention [25]. In this study, 96 teachers (82.8%) reported proficiency in detecting such signs, contrasting with 13 (11.2%) who acknowledged limited recognition capabilities, while seven (6.0%) responded, “don’t know/no answer”. Crucially, 112 participants (96.6%) observed temporal fluctuations in MF throughout the academic year, with only two (1.7%) reporting stable levels. The recognition of this cyclical variability represents a key consideration when periodizing fatigue mitigation strategies across the academic calendar, thereby enabling individualized interventions.

### 4.2. Causes of Mental Fatigue in Students

Understanding the causes of MF in adolescent students will allow PE teachers to develop and optimize general, activity-based strategies for application within the school environment [19]. Among the majority of participating PE teachers (77.6% to 87.9%), several key causes were identified, including examinations, prolonged sitting time at school, low physical fitness, limited extracurricular PA, and extended school hours without opportunities for PA. Notably, most of these factors are associated with PA, sedentarism, or physical fitness. This indicates that PE practitioners recognize the influence of PA on MF, specifically that low PA or fitness levels may precipitate or exacerbate MF in students. These findings underscore the critical need to promote active lifestyles among adolescents [13] and to leverage the benefits of PA, such as improved cognitive function, stress reduction, and enhanced emotional resilience in mitigating MF [14]. Concurrently, a significant proportion of respondents (48.3% to 61.2%) agreed that specific school characteristics, including demanding schedules, homework load, teaching methodologies, and academic pressure, also contribute to MF. These academic stressors align with the models of self-regulatory resource depletion [26] and student self-reports of cognitive overload [27], suggesting that effective intervention requires a comprehensive, school-wide approach.

### 4.3. Consequences of Mental Fatigue in Students

Building upon the identified causes, the significant repercussions of MF in adolescent students highlight the critical need for teacher awareness and the implementation of effective mitigation strategies [17]. Consequently, investigating PE teachers’ perceptions on this issue is a necessary step to inform the development of targeted professional training. In this study, the vast majority of participants perceived significant negative impacts, a finding that is fundamental for promoting evidence-based interventions. Specifically, nearly 95% of respondents identified increased irritability and impatience as the most widely endorsed consequence, which often leads to behavioral problems [28]. Furthermore, over 80% of PE teachers associated MF with impaired cognitive capacity, worsened academic performance, a deteriorated emotional state, increased stress, heightened anxiety, and reduced motivation. These perceptions align with meta-analytic evidence demonstrating that acute MF impairs performance on executive function tasks [29] and fine-motor performance [30]. Within PE contexts, these deficits manifest as slower skill acquisition and diminished enjoyment among students [31], potentially amplifying a negative cycle of inactivity and poor academic outcomes [32]. Finally, PE teachers also highlighted other relevant consequences, including diminished sleep quality, strained interpersonal relationships, and increased physical fatigue, all of which are areas that could be ameliorated through structured PA [33].

### 4.4. The Role of Physical Activity in Mental Fatigue Attenuation

Given the significant repercussions of MF in adolescent students, targeted interventions to mitigate its effects are essential. The findings demonstrate strong consensus among PE teachers regarding the modifiability of this condition. Specifically, an overwhelming majority (91.4%; *n* = 106) affirmed that MF is amenable to positive influence, while a small minority denied this possibility (1.7%; *n* = 2) or remained undecided (6.9%; *n* = 8). Furthermore, the vast majority of the participants (96) indicated that PA is a viable pathway for influencing MF, with a near-total consensus against the contrary (only one disagreement and three “don’t know/no answer” responses). These perceptions corroborate existing empirical evidence on the psychological benefits derived from regular PA [13,14]. Finally, 93 teachers confirmed a belief that improving students’ physical fitness would reduce their perceived MF, which was a view held by the strong majority compared to a small number of dissenters (three) and those who were uncertain (four). This suggests that adequate physical fitness, likely attained through sustained PA, is perceived as a critical factor in reducing MF [14].

Regarding the practical implementation, the majority of teachers (69.0%; *n* = 80) reported actively applying strategies to reduce student MF, while a significant proportion (21.6%; *n* = 25) did not employ such methods, and the minority (7.8%; *n* = 9) were unsure. This disparity is highly relevant; despite recognizing the importance and benefits of these strategies, the number of teachers not implementing them remains considerable. This suggests that future research should investigate the specific barriers hindering the application of school-based, PA-oriented strategies for reducing mental health issues in students. Indeed, despite high perceived efficacy amongst the staff, 31.4% of teachers (combining those who do not apply strategies and those who are unsure) have not yet adopted MF mitigation techniques. Commonly reported barriers in the literature include a lack of institutional support, rigid curricular timetables, and insufficient teacher training [34,35]. Therefore, the targeted professional development programs and more flexible scheduling may be essential to bridge this gap between positive attitudes and routine pedagogical practice.

Notably, PE teachers rated some scholar PA strategies, such as active recess, classroom movement breaks, and active lessons, as the most effective countermeasures against MF. In contrast, high-intensity PA was perceived as the least effective strategy. This perception aligns with physiological evidence indicating that moderate PA optimally facilitates cerebral reoxygenation and upregulates prefrontal dopamine without inducing additional fatigue [36]. Although high-intensity exercise is beneficial for cardiovascular fitness, it can acutely exacerbate the strain on executive neural networks already depleted by cognitive tasks [37], which may explain why teachers placed less value on its application for this specific purpose. Consequently, while the PE teachers’ consensus is physiologically plausible, the comparative efficacy of high-intensity PA programs for MF mitigation remains empirically underexplored, underscoring a clear need for further investigation.

### 4.5. Limitations and Future Research

This study acknowledges three primary limitations. First, the reliance on PE teacher self-reports introduces potential estimation bias regarding actual MF prevalence, compounded by the absence of objective neurofunctional measures (e.g., psychomotor vigilance tasks or EEG). Second, the exclusive focus on PE teachers’ perspectives neglects triangulation with student experiences or administrative insights, resulting in a teacher-centric understanding of MF antecedents and consequences. Third, the findings were derived exclusively from the Spanish educational context. The differences in educational policies, curriculum structures, and cultural factors across countries may influence the implementation of PE. Therefore, caution should be exercised when generalizing these findings to other international contexts. Finally, the use of chain sampling may limit the representativeness of the sample, and the findings should therefore be interpreted with caution when generalizing to the broader population. Future research should be focused on (a) implementing longitudinal designs integrating subjective appraisals with objective biometrics to validate educator perceptions; (b) conducting controlled trials comparing recess-based movement breaks, classroom-embedded PA microdosing, and high-intensity interventions for differential impacts on MF and academic outcomes; and (c) examining the interactions between individual moderators (e.g., sleep hygiene or mental health status) and institutional policies to develop school-specific adaptation frameworks. Such methodological advancements would establish evidence-based protocols for periodizing fatigue mitigation strategies across academic calendars.

## 5. Conclusions

Spanish PE teachers demonstrate clear recognition of student MF as both a pervasive academic impediment and temporally dynamic phenomenon across the academic year. The primary attribution centers on four systemic factors: hyper-compressed timetables, excessive homework loads, passive knowledge transfer methodologies, and performance-oriented academic pressures. The teachers identify moderate, curriculum-embedded PA interventions, particularly structured active recess, brief in-class movement breaks, and physically integrated lessons, as optimal evidence-based countermeasures. The substantial implementation gap between the perceived efficacy and practical adoption underscores the critical need for institutional scaffolding through policy reforms, flexible scheduling mechanisms, and competency-focused professional development to transform pedagogical awareness into sustained practice. Furthermore, fostering collaborative working groups that involve other members of the educational community or neighborhood can enhance implementation and support sustainable strategies to mitigate student mental fatigue.

## 6. Practical Applications

For practical implementation in compulsory secondary education, we propose operationalizing these findings through scheduled classroom interventions: embedding teacher-facilitated 3–5 min movement breaks after 45 min theoretical sessions to counteract cognitive saturation; transforming recess into supervised active zones with non-competitive stations (e.g., dynamic math problem-solving via court-marking navigation and literature-based movement sequences) targeting ≥70% of student engagement; redesigning homework protocols via the 20-20-20 recovery rule (20 s stretching/visual resets per 20 min of academic work); and systematically training teachers in fatigue-sign recognition and age-appropriate activity prescription, all of which should be integrated through departmental coordination to overcome the implementation barriers while aligning with the teachers’ evidence-based preferences for curriculum-embedded PA solutions.

## Data Availability

The data presented in this study are available on request from the corresponding author. The data are not publicly available due to ethical restrictions.

## Abbreviations

The following abbreviations are used in this manuscript:

PE	Physical education
PA	Physical activity
MF	Mental fatigue
